# Landmarks in Developmental Biology and Evolution

**DOI:** 10.2174/138920212800793320

**Published:** 2012-06

**Authors:** AJ Durston

This mini hot topic is the first in a series ‘'Landmarks in Developmental Biology and Evolution,' intended to celebrate some recent landmarks and share excitement in Developmental Biology and Evolution. The ideas and discoveries here are by definition not mainstream. They break the mould by sheer unexpected serendipitous inspiration and may well upset the establishment. They can change history. Landmark discoveries are only made by going against the flow.

What is exciting about science? Not impact factor nor translational importance (though the latter is important). Nature is beautiful. Who looking at a beautiful landscape, the movement of an eel, or the development of an embryo, could fail to feel a sense of awe? The same is true with the mechanisms underlying natural phenomena and the inspirational ideas and investigations that elucidate them. Darwin’s theory of natural selection, Spemann’s organiser, the Operon concept, E.B. Lewis’s analysis of the Bithorax complex, Newport and Kirschner’s MBT. Magic! The whole of embryonic development is a clear case in point. Many of us in this field have been inspired to start our careers by one or other of the findings above. The funding agencies seem to have lost sight of this.

Why, then, one is not filled with joy by the average scientific meeting? This may be something to do with dogma. At a certain point, a scientific idea gains in respectability and sometimes credibility to the point that it is widely accepted. A natural part of the scientific process- but it has a dark side. Dogma can generate totalitarianism and stifle creativity. Young scientists are discouraged from pursuing original radical ideas that challenge dogma. Obedience is required. Science politics kicks in, with impact factors, harsh, non objective peer reviews and ‘people who represent a field’. Attention focuses on the detailed and less interesting aspects instead of pushing boundaries. This is pernicious. It spells the death of science, which becomes a religion and tediously boring. The development of a scientific area is often like a comet. An initial brilliant discovery and a diminishing tail of sparks. Those who challenge dogma become renegades- until their ideas become the new dogma.

What is to be done? The purpose of this ‘mini hot topic’ series is to make a modest start in giving the mavericks a voice. We give exposure to and celebrate recent exciting radical ideas that challenge dogma and have the potential to change history. Some already have. Of course, they, like dogma, can be wrong. The ‘mad scientist’ is familiar. But if they are elegant, like nature, we should suspect there is truth. At any rate, they are fun and stimulate the imagination. Some of our ‘Mini Hot Topic issues’ will contain my personal choices, reflecting ideas and discoveries that have fired my own imagination. Others will be guest edited by inspiring colleagues

## DESIGNING THE BODYPLAN

The specific purpose of this first ‘Mini Hot Topic’ is to present a small collection of highly original reviews in an interesting and intellectually stimulating area. The area is ‘Designing The Bodyplan’- the mechanisms that generate shape and structure in the developing embryo. The area is further restricted in this issue to mechanisms involved in generating the animal’s main body axis. Within this area, we concentrate on unexpected developments that have recently opened new perspectives. We avoid the very well known findings- like discovery of the vertebrate somitogenesis clock, the standard ideas on Hox genes and their collinearity and the Drosophila segmentation gene hierarchy that have now become dogma and we think, do not need to be reviewed again. We choose topics that we hope will surprise the reader.

These are:

### Kees Weijer

(Dundee), with his novel discovery that positive and negative chemotaxis involving at least 3 different ligand receptor systems mediates directional cell and tissue movements that are a key part of the morphogenetic mechanism for building the vertebrate main body axis during gastrulation. This discovery came at a time when the gastrulation field was lacking in inspiration and heavily bogged down in extracellular matrix and cell adhesion. This field was mostly going nowhere, despite a couple of shafts of light in the gloom like the inspiring work of Ray Keller. This remains a difficult field but there is now light at the end of the tunnel, with a mechanism that can credibly sensitively guide mass cell movements. To be fair, this was not the very first report of chemotaxis in vertebrate development. There were a couple of previous studies. And Wejjer himself has made amazing discoveries about chemotaxis in Dictyostelium. But this is the first serious and inspiring investigation. 

### Eddy McGlinn

(Monash), with her novel discovery that microRNA’s mediate an aspect of Hox collinearity. She and her colleagues in Cliff Tabin’s lab. at Harvard discovered that posterior prevalence- the ability of more posterior Hox genes to dominate more anterior ones can involve these novel molecules. This is an example of the intelligent use of a new discovery to elucidate a subtle developmental function. Most papers on the recently discovered miRNA’s lack anything interesting about their biological function. The importance of posterior prevalence has been underestimated and it is refreshing to see this new investigation of it. This finding also challenges the unspoken prejudice that the important features of Hox regulation are transcriptional and only to be elucidated by characterizing enhancers and global control regions. 

### Joost Woltering

(Geneva), with his novel discovery that the deregionalized body plan of snakes is due to an altered dowstream response to the axial Hox pattern. This is surprising considering the previous evidence that the functions of Hox genes are highly conserved in evolution and the previous prevailing assumption that changes in Hox expression patterns underlie the evolutionary changes in the snake's body plan. Woltering's finding shows that similar Hox codes can specify strikingly different axial positions in different vertebrates. Woltering’s paper strongly influenced further thinking in the field and was confirmed by later publications from major laboratories. I have asked the young scientist who made the original conceptual leap to write this review. 

### Myself

(Leiden). I have taken the liberty of including one of our own ideas. Time space translation: a Hox mechanism for vertebrate A_P patterning. Timed sequences of events are used to generate spatial patterns. This is worked out in relation to genesis of the vertebrate embryo’s main axial Hox pattern from a temporally collinear sequence of Hox expression. The same principle applies to vertebrate developmental mechanisms ranging from somitogenesis to limb development. I challenge dogma ideas about Hox temporal collinearity and axial patterning and touch on the importance of posterior prevalence.

